# Deep Sequencing Reveals a Divergent *Ugandan cassava brown streak virus* Isolate from Malawi

**DOI:** 10.1128/genomeA.00818-17

**Published:** 2017-08-17

**Authors:** Willard Mbewe, Stephan Winter, Settumba Mukasa, Fred Tairo, Peter Sseruwagi, Joseph Ndunguru, Siobain Duffy

**Affiliations:** a Department of Ecology, Evolution and Natural Resources, Rutgers University, New Brunswick, New Jersey, USA; b School of Agricultural Science, Makerere University, Kampala, Uganda; c DSMZ Plant Virus Department, Leibniz Institute, Braunschweig, Germany; d Mikocheni Agricultural Research Institute, Dar es Salaam, Tanzania

## Abstract

Illumina sequencing of RNA from a cassava cutting from northern Malawi produced a genome of *Ugandan cassava brown streak virus* (UCBSV-MW-NB7_2013). Sequence comparisons revealed stronger similarity to an isolate from nearby Tanzania (93.4% pairwise nucleotide identity) than to those previously reported from Malawi (86.9 to 87.0%).

## GENOME ANNOUNCEMENT

*Ugandan cassava brown streak virus* (UCBSV) is one of two distinct ipomoviruses (family: *Potyviridae*) that cause the devastating cassava brown streak disease (CBSD) (1, 2) of cassava (*Manihot esculenta*) in eastern and central Africa. In Malawi in 2013, cassava cuttings with conspicuous symptoms of CBSD were collected, transferred to the DSMZ Plant Virus Department, and rooted and grown under glasshouse conditions optimized for cassava and viruses. Virus assays were conducted to identify and discriminate between CBSV and UCBSV essentially following the protocol of Mohammed et al. (3). Total RNA of a cassava cutting from Nkhata Bay District was extracted using an RNeasy Plant minikit (Qiagen, Germany), and rRNA was removed using a RiboMinus plant kit (Invitrogen). Following random cDNA synthesis and second-strand synthesis with random octamer primers, an Illumina library was prepared using a Nextera XT library kit and subsequently run on a MiSeq instrument as paired-end reads (2 × 301). In total, 1,241,000 reads were generated. *De novo* assembly was conducted in both CLC Workbench and Geneious version 10.0.5 (4). A well-supported sequence contig was obtained (average 300× coverage) that showed highest nucleotide identity with UCBSV. This 9,070-nucleotide sequence, UCBSV-MW-NB7_2013, was compared to all complete UCBSV genome sequences available in GenBank (Tanzania, FJ039520; Kenya, FN433931, FN433930, KR911721, KR911722, KR911723, KR911724, KR911725, KR911726, and KR911727; Uganda, HM181930, FJ185044, HG96522, and FN434109; and Malawi, FN433932 and FN433933) using MEGA version 7 (5).

Pairwise nucleotide sequence similarity of UCBSV-MW-NB7_2013 with UCBSV genome sequences showed 87.9 to 93.4% nucleotide identities, with the highest identity to the sequence of the UCBSV MLB3 isolate (FJ039520) from Tanzania. The amino acid sequence of both polyproteins was 95.8% identical. UCBSV-MW-NB7_2013 was more distantly related to previously characterized Malawian isolates, with a maximum percentage identity of 87.0% with isolate Ma 43 (FN433933). The 230 nucleotides of the 3′ untranslated region (UTR) had high identity to extant UCBSV genomes (97% identity to Ma 43, with the terminal 52 nucleotides identical). The terminal 8 nucleotides of the 5′ UTR of our UCBSV isolate (TCACATAC) were very divergent compared to all previously sequenced UCBSVs, which is most probably artifactual due to the low coverage at the ends of the contig. However, newly sequenced full UCBSV genomes from Kenya have revealed unexpected diversity in the 5′ UTR (6), and further sequencing of UCBSV isolates could reveal that the 5′ terminus is more variable than previously thought.

A BLAST search for the UCBSV-MW-NB7_2013 sequence against the GenBank nonredundant database showed the top two hits to be the Tanzanian full genome and the only other UCBSV sequence from Tanzania, the partial genome sequence KR108839. The similarities between this isolate from northern Malawi and UCBSV from Tanzania suggest that it may have been introduced from Tanzania, since is not as closely related to CBSD-causing viruses previously detected in Malawi.

### Accession number(s).

The whole-genome sequence of UCBSV-MW-NB7_2013 has been deposited in DDBJ/ENA/GenBank under the accession no. MF379362.
